# Understanding the Interplay Between Health Disparities and Epigenomics

**DOI:** 10.3389/fgene.2020.00903

**Published:** 2020-08-20

**Authors:** Viviana J. Mancilla, Noah C. Peeri, Talisa Silzer, Riyaz Basha, Martha Felini, Harlan P. Jones, Nicole Phillips, Meng-Hua Tao, Srikantha Thyagarajan, Jamboor K. Vishwanatha

**Affiliations:** ^1^Department of Microbiology, Immunology and Genetics, Graduate School of Biomedical Sciences, University of North Texas Health Science Center, Fort Worth, TX, United States; ^2^Department of Biostatistics and Epidemiology, School of Public Health, University of North Texas Health Science Center, Fort Worth, TX, United States; ^3^Department of Pediatrics, Texas College of Osteopathic Medicine, University of North Texas Health Science Center, Fort Worth, TX, United States; ^4^Texas Center for Health Disparities, University of North Texas Health Science Center, Fort Worth, TX, United States

**Keywords:** epigenetics, cancer, health disparities, chronic disease, social determinants of health

## Abstract

Social epigenomics has emerged as an integrative field of research focused on identification of socio-environmental factors, their influence on human biology through epigenomic modifications, and how they contribute to current health disparities. Several health disparities studies have been published using genetic-based approaches; however, increasing accessibility and affordability of molecular technologies have allowed for an in-depth investigation of the influence of external factors on epigenetic modifications (e.g., DNA methylation, micro-RNA expression). Currently, research is focused on epigenetic changes in response to environment, as well as targeted epigenetic therapies and environmental/social strategies for potentially minimizing certain health disparities. Here, we will review recent findings in this field pertaining to conditions and diseases over life span encompassing prenatal to adult stages.

## Introduction

Social epigenomics is defined as the study of how social experiences affect our genes and biology. Though social epigenomics is a relatively new area of research, studies exploring the individual and combinatorial influence of social, environmental, and genetic factors on health have become increasingly abundant. Social epigenomics is uniquely positioned at the intersection of population health and precision medicine, allowing us to understand how exposure to social and environmental stressors modifies the way in which genes are expressed and ultimately alter our risk for disease. This area of research is important when it comes to understanding the biological effects of environmental (e.g., food availability, pollution, green space, etc.) or social stressors (e.g., abuse, socioeconomic stress, etc.) and how they contribute to the rising health disparities commonly affecting minority communities; however, health disparities within minority populations have not been well addressed using epigenetic approaches. This space has gained increasing interest over recent years, as reflected by the $26.2 million-dollar National Institutes of Health (NIH) initiative entitled Social Epigenomics Research Focused on Minority Health and Health Disparities that was introduced in 2017.

Several recent and exciting discoveries have been made in the area of social epigenomics, which have allowed researchers to slowly disentangle the roles that social, environmental, and genetic factors may play on health and disease risk. The areas reviewed here are: (1) key social determinants of health, (2) common epigenetic mechanisms that affect human biology, (3) intersection of social determinants and epigenetics over the human life span, and (4) challenges and current limitations of social epigenomic studies.

## Key Social Determinants of Health

Social determinants of health can be viewed as “conditions in the environments in which people are born, live, learn, work, play, worship, and age”; these conditions influence health outcomes throughout the life course and in a multitude of ways (Institute of Medicine et al., 2006; Office of Disease Prevention and Health Promotion, 2020). Many of these determinants are intertwined, revealing a complex web of interconnected relationships, with both a direct and indirect impact on population health. Several key factors encompass the broad definition of social determinants including family and neighborhood effects, exposure to chronic stress, socioeconomic status (SES), educational attainment, access to health care, job availability, and exposure to crime and violence (Braveman and Gottlieb, 2014). Neighborhood effects can refer to the physical environment where an individual or family lives, or the social environment, which can be defined by a vast state of relationships between individuals within a neighborhood (Chaix, 2009). With respect to the physical environment, in urban and disadvantaged neighborhoods, epidemiologic studies consistently report that exposure to pollutants and allergens leads to worsening lung health (discussed in further detail in the early-life section) (Braveman and Gottlieb, 2014). Furthermore, in disadvantaged neighborhoods, there tends to be higher availability of alcohol, tobacco, fast-food restaurants, combined with lower availability of healthy food options (i.e., food deserts) and areas for recreation which lead to worse health outcomes (Braveman and Gottlieb, 2014). This leads to increased intake of unhealthy foods, less opportunity for physical activity, and as a result of alcohol consumption, an increased risk for alcohol-related traumatic injury (Braveman and Gottlieb, 2014). Furthermore, increased exposure to chronic stress in these populations has been associated with epigenetic changes and has been theorized, in part, due to a lack of social and familial support networks for coping (Cunliffe, 2016). Individual-level and neighborhood-level effects are closely intertwined and likely reciprocally influence one another. Disease is naturally an individual-level event, and as such, neighborhood-level influences exert their effects through behaviors and biologic processes (Diez Roux, 2001). For example, the availability (or lack thereof) of healthy food options in a neighborhood may drive individual-level choices for nutrition, leading to increased intake of polyunsaturated fats which may generate mutagenic free radical and oxidative stress (Bartsch and Nair, 2004; Alegría-Torres et al., 2011). This, in turn, may lead to epigenetic alterations that affect downstream biological processes, ultimately manifesting in disease (e.g., oxidative stress-induced lung cancer) (Lawless et al., 2009), though the underlying biology is often much more complex. In the context of neighborhood effects, home-level effects present another domain within which disparities can occur. The impact of poor housing conditions on health has been extensively studied (Krieger and Higgins, 2002); for example, the impact of indoor air quality on the risk or exacerbation of asthma. Studies have found that the onset and severity of asthma may be affected by interactions between the physical environment and epigenetic factors (e.g., *ADRB2 5’-UTR* methylation) (Fu et al., 2012). As a result of differences in the social determinants of health between populations, health disparities are commonplace. One proposed mechanism connecting these determinants with health outcomes is epigenetics. However, research focusing on the interplay between epigenetic (or epigenomic) changes and these determinants is limited. Epigenetics by no means fully explains these disparities, though it provides insight into the interplay between the environment and genetics in the context of disease risk, pathology, and severity.

Differences in the distribution of these social determinants are evident in the United States. Health disparities exist in many forms, including higher rates of chronic disease and premature death among minority racial and ethnic groups when compared to Caucasians, although the trends are not universal (National Academies of Sciences et al., 2017). Interestingly, in some minority groups, for example, Hispanic immigrants, better health outcomes are seen when compared to non-Hispanic whites (known as the “immigrant paradox”); however, this association diminishes as time spent in the United States increases (National Academies of Sciences et al., 2017). A stepwise socioeconomic gradient has recently been observed in the United States, overall and within racial and ethnic groups, with improvements in health increasing as social advantage increases (measured by SES); those among the most affluent and educated have the best health outcomes, while those with moderate and low income have worse health outcomes (Braveman et al., 2011). These social determinants may play a significant role in influencing overall health and disease risk potentially *via* epigenetic modifications affecting downstream molecular processes.

## Common Epigenetic Mechanisms That Affect Human Biology

Since the mid-20th century when Waddington first introduced the concept of epigenetics, its definition has transformed, reflecting our increase in understanding of the molecular mechanisms underlying human biology from conception to death; for a recent review on the history of epigenetics terminology and findings, refer to Felsenfeld (2014). Epigenetics is presently defined as mitotically inheritable modifications to DNA that do not directly alter the sequence. These modifications can be *de novo* or inherited *via* genetic imprinting. Though epigenetic signatures are well known for their role in determining cell fate, epigenetic marks can also change in response to genetic and environmental factors over time, resulting in subsequent biological changes that may be tissue or cell type specific. The mechanisms responsible for regulating these modifications are capable of altering gene expression at multiple levels (e.g., transcriptional, post-transcriptional, translational, and post-translational).

### DNA Methylation

DNA methylation is one of the most extensively studied epigenetic modifications. Several different types of DNA methylation exist [e.g., 5-methylcytosine (5-mC), 5-hydroxymethylcytosine (5-hmC)]; however, here, we will only review the most common form, 5-mC. This modification is characterized by the addition of methyl groups to the fifth position carbon of cytosine nucleotides; often, these cytosines are located adjacent to guanine (this position is termed a “CpG site”). Of the different regions within the human genome, gene promoters are typically enriched for C-G dinucleotides, yielding regions called “CpG islands.” DNA methyltransferases (DNMTs) transfer donated methyl groups from S-Adenosyl Methionine (SAM) complexes to target sites; DNMT1 enzymes serve to maintain pre-existing sites, while DNMT3A and DNMT3B transferases serve to establish *de novo* methylation marks. Though methylation within promoter regions is often negatively correlated, methylation within the gene body has been found to be positively correlated with gene expression, suggesting site-specific effects (Jones et al., 2015). Further, DNA methylation patterns are known to be tissue specific and highly conserved; interestingly, this conservation is thought to be controlled, in part, by DNA sequence at transcription factor binding sites (Zhou et al., 2017). For a recent review on the effects of DNA methylation on transcription factor binding, refer to Héberlé and Bardet (2019).

### Histone Modifications

Eukaryotic chromatin structure consists of highly condensed, repeating “bead-like” structures called nucleosomes, which are units composed of approximately 147 base pairs of DNA wound around an octomeric histone core (consisting of two copies each of H2A, H2B, H3, and H4 histones) with linker histones (H1) connecting octamers of neighboring nucleosomes. Regulation of gene expression at the chromatin level can occur through chromatin remodeling (e.g., Swi/Snf complexes) and/or addition of covalent modifications such as acetyl, methyl, ubiquitin, phosphate, and biotin groups to basic (e.g., lysine, arginine) residues embedded in exposed histone N-terminal tails. As histone proteins play a significant role in DNA packaging, alterations to side chains can impact transcriptional activation/repression and efficiency of DNA repair mechanisms (Shen et al., 2006). Addition and removal of these modifications are regulated by enzymes termed “writers” and “erasers,” respectively, while the downstream effects of these modifications on gene expression are interpreted and dictated by protein factors termed “readers” (Gillette and Hill, 2015).

The most extensively studied histone modifications are acetylation and methylation, which have both direct and indirect effects on transcription (Bannister and Kouzarides, 2011). The action of each modification is regulated by enzymes termed “readers,” “writers,” and “erasers.” Acetylation status is primarily regulated by histone acetyltransferases (HATs) and histone deacetylases (HDACs). Acetyl groups added to lysine residues by HATs are typically read by small bromodomain proteins and allow for neutralization of the positive charge, leading to subsequent weakening of histone–DNA interactions and relaxation of chromatin structure (Marmorstein and Zhou, 2014). This provides regulatory and transcription factors with propensity for DNA to bind and enhance transcription. Though acetylation occurs throughout multiple regions of the genome, promoter and enhancer regions are often enriched with histone acetylation marks (Bannister and Kouzarides, 2011). Conversely, histone methylation status is primarily regulated by histone methyltransferases (HMTs) and histone demethylases (HDMs). Methyl groups are donated by SAM and transferred by HMTs to H3 and H4 arginine and lysine side chains, which are then read by a variety of different protein readers (depending on location and number of methyl groups) (Musselman et al., 2012). These methyl marks promote both transcriptional repression and activation, depending on the genomic context, by regulating DNA supercoiling (Barski et al., 2007).

The biological consequences of these modifications are further complicated, as there is evidence to suggest that chromatin structure varies based on type, location, and number of modifications, including cross-talk effects among different modification types (Molina-Serrano et al., 2013). For example, trimethylation of H3 lysine residues (H3K4me3) has been shown to occur only in the absence of H3 arginine dimethylation (H3R2me2a) (Molina-Serrano et al., 2013). Though histone modifications are important for various developmental processes, dysregulation or changes in the patterns of these epigenomic marks have also been implicated in aging and several diseases.

### Micro-RNAs and Long Non-coding RNAs

Both micro-RNAs (miRNAs) and long non-coding RNAs (LncRNAs) play important roles in regulating gene expression. MiRNAs range in size from approximately 22 to 26 nucleotides in length. They contain 2–7 base pairs of complementary sequence (termed “seed sequence”) that bind to a stretch of nucleotides, often in the 3’-untranslated region (3’-UTR) of the target mRNA [though ubiquitous binding to other gene regions has been observed (O’Brien et al., 2018)], leading to either degradation of the transcript or inhibition of translation. Often, members of the same miRNA families, or rather those with similar seed sequences, target the same gene families or biological pathways (Backes et al., 2018). LncRNAs are >200 nucleotides in length and can influence gene expression through chromatin remodeling as well as interaction with transcriptional and post-transcriptional processing machineries (Cao, 2014).

## Intersection of Social Determinants and Epigenetics Over the Human Life Span

Epigenetic modifications are dynamic in nature, and their patterns have been observed to change in response to environmental and psychosocial factors and have also been implicated in several disease states. These changes can occur at the level of chromatin, DNA, and RNA as highlighted in the previous section. These modifications also exhibit high levels of plasticity throughout the course of the human life span, though this plastic nature inevitably slows over time. Here, we briefly review recent findings implicating the intersection of social determinants and epigenetics, though this review is not exhaustive.

**FIGURE 1:**
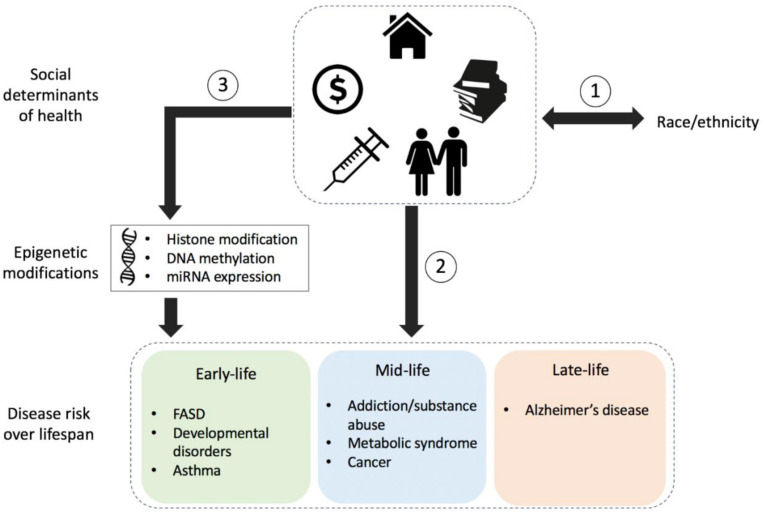
Schematic detailing the influence of socio-environmental factors on health and risk for disease. (1) Race/ethnicity is often associated with an individual’s social determinants of health including local neighborhood, social environment, education levels, socioeconomic status, and access to health care. (2) The social determinants of health can influence the risk for disease throughout the lifetime, regardless of genetics; however, (3) they can also influence epigenetic modifications such as those to histones, DNA, or micro-RNA (miRNA), thereby influencing biological functions that have downstream effects on health and disease susceptibility throughout the life span. Conditions and/or diseases within the colored boxes indicate socio-epigenomic links at different stages throughout the human life span: early-life, mid-life, and late-life; conditions detailed here are not exhaustive. FASD, fetal alcohol spectrum disorder.

Figure 1 highlights the complex nature of health disparities over the human life span. Race/ethnicity or underlying ancestral genetic variation may be associated with risk for particular conditions but may also drive epigenetic alterations (not shown) that later contribute to disease risk and/or pathology. Regardless of genetic factors, social determinants of health, in combination with a variety of environmental exposures over time, may also contribute to disease risk.

### Early Life

#### Prenatal Alcohol Exposure

Prenatal exposure to toxic agents, such as alcohol, radiation, environmental pollution, and maternal infections, can lead to a range of adverse developmental outcomes. *In utero*-derived disorders can be difficult to discuss and diagnose as mothers can become focal points of blame, sometimes leading to dishonest or reserved discussions between the mother and health care provider. For example, exposure to alcohol early in pregnancy can have the most detrimental effects. However, most mothers who drink early in pregnancy often do not know that they are pregnant at the time or may not report drinking while being pregnant due to social consequences. This prevents proper and timely diagnosis of a child who may be showing early signs of fetal alcohol spectrum disorder (FASD), classically characterized by neurocognitive deficiencies, impaired self-regulation, and adaptive function; these characteristics can also persist into adulthood (Lussier et al., 2017).

Fetal alcohol spectrum disorder was first described by Jones and Smith (1973) in 1973 and broadly includes several neurodevelopmental disorders described by physical, cognitive, memory, behavioral, and learning difficulties. Hallmarks of FASD include congenital malformations, deformities, chromosomal abnormalities, and mental/behavioral conditions. These issues persist throughout the afflicted child’s lifetime which is often shortened and includes premature onset of chronic disease. Although FASD may not affect all who are prenatally exposed to alcohol, worldwide prevalence is estimated to be approximately 1–5% (May et al., 2009; Lange et al., 2017). Further breakdown of FASD demographics within developed countries highlights a major health disparity, whereby groups most affected tend to be of lower income and/or belong to a minority. Notably, Inuit and Native Americans hold the highest rate of FASD followed by African Americans (Tenkku et al., 2009). Interestingly, Hispanic females vary in risk depending on origin of birth. According to a review by Bakhireva et al. (2009), Latinas born in the United States were shown to be more likely to drink when compared to Latinas born outside of the country due to differences in cultural norms. Several explanations for this observation have been proposed including higher education and income and loss of traditional community leading to an increased rate of unhealthy behaviors. On the other hand, immigrants may also experience increased alcohol consumption due to the stress of assimilation. Current estimates of FASD occurrence are believed to be an underestimation due to several reasons including the fact that assessment relies on mothers to report drinking, which again is shrouded in stigma, thus delaying the diagnostic process. A recent review describes that although individuals with higher SES may drink about as much if not more than individuals with lower SES, they do not experience as many of the negative outcomes of alcohol consumption (Collins, 2016). It is suggested that although further studies are required to determine the mechanisms, the outcomes may be moderated by race, ethnicity, and gender.

Recent rodent models have identified altered DNA methylation patterns in protocadherin genes and deregulation of genes possibly due to prenatal alcohol exposure with follow-up buccal swabs from children showing similar patterns (Laufer et al., 2017). For example, upon alcohol exposure, embryo growth was restricted due to hypomethylation of the regulatory region of the *H19* gene present in the human placenta, suggesting the incidence of genomic imprinting (Haycock and Ramsay, 2009).

In response to the lack of investigation into the social impact of FASD diagnosis, efforts have been made to promote studies in this field of research. Translating to the Community (T2C) is an initiative established by researchers at the University of Manitoba and is the first Canadian social epigenetic biobank for Aboriginal communities that are known to have a disproportionate prevalence of FASD (Elias et al., 2018). The biobank is focused on the collection of biological samples (e.g., saliva, blood), social-contextual health-survey data, and clinical data in order to identify risk factors, social and biological pathways implicated in FASD. Efforts such as T2C may allow for a better understanding of the biological basis of FASD and identification of environmental and/or societal factors that increase the risk for FASD and have led to current disparities. Biobanks have historically lacked diversity; however, T2C, All of Us, and the United Kingdom Biobank are some of the organizations working toward inclusion (Collins and Varmus, 2015; Bycroft et al., 2018).

#### Micro-RNAs, Stress, and Pregnancy

MiRNAs are small non-coding segments of RNA which modulate gene expression by inhibiting translation. Expression of miRNAs is regulated by RNA polymerase II (similar to mRNA), and they are transported from cell to cell *via* exosomes (Hayashi and Hoffman, 2017). Circulating miRNAs in biofluid are believed to be a potential biomarker for a number of conditions (Gilad et al., 2008). Differential miRNA expression has been observed between tumors and normal tissue in multiple cancer types (Lu et al., 2005). Interestingly, certain maternally derived miRNAs (e.g., MIR517A) have been shown to be of placental origin, circulate within the mother’s plasma, and are cleared shortly following delivery (Luo et al., 2009). The expression levels of miRNA cluster C14MC and other pregnancy-specific miRNAs fluctuate over a normal pregnancy and throughout fetal development (Morales-Prieto et al., 2013). However, changes in miRNA expression have also been described throughout several pregnancy complications including preeclampsia, preterm birth, and gestational diabetes (Ospina-Prieto et al., 2016; Cao et al., 2017; Fallen et al., 2018). During pregnancy, miRNAs regulate multiple targets involved in immune suppression, tumor regulation, and protein trafficking.

The expression of pregnancy-related miRNAs is susceptible to environmental changes including psychological and physical stress, which may affect the development of the child or lead to disease onset. Diseases triggered by stress are typically multifactorial in nature and can vary in severity due to a combinatorial effect of gestational stage, maternal age, and race. As studies have shown, miRNA expression fluctuates over the lifetime in response to environmental stimuli. MiRNA profiling has the potential to serve as a less invasive method of fetal monitoring compared to other commonly used methods such as amniocentesis.

The Maternal and Developmental Risks from Environmental and Social Stressors (MADRES) cohort was created to help understand health disparities in low-income females in Los Angeles by collecting biosamples and information over the course of pregnancy and early life (Bastain et al., 2019). This center focuses on individual and cumulative factors (e.g., stress, environmental toxin exposure) involved in childhood obesity and excess pregnancy-related weight gain and postpartum retention. Information and biological samples from mothers and infants are collected during pregnancy, at birth, and throughout the first year of the child’s life. The collected information/samples include: questionnaires regarding household size, education, income, access to health care, proximity to freeways, and exposure to industry-derived toxins to assist in determining extrinsic stress; biospecimens including blood, urine, hair, nail clippings, feces, and saliva from pregnant mothers prior to and during birth; additional specimens collected at birth include umbilical cord blood, newborn blood, and placental tissue. The center assesses the health of both the mother and child using several different biological assays, including DNA methylation and metal exposure levels. The questionnaires provide measures of several levels of stress (Perceived Stress Scale, Prenatal Distress Questionnaire, and Center for Epidemiologic Studies). Studies utilizing information collected from this cohort highlighted the importance of collecting longitudinal data to assess the health of both mother and baby through the course of pregnancy, particularly in minority communities, to identify the factors that may be contributing to health disparities (Bastain et al., 2019).

#### Asthma Disparities

Asthma is one of the most common chronic diseases in children. It is often diagnosed in children who live in inner-city areas, neighborhoods near areas of high automotive traffic and emissions, or who attend day-care centers in early childhood (Ochoa Sangrador and Vázquez Blanco, 2018). Asthma is characterized by difficulty in breathing due to respiratory airway swelling and inflammation. Several factors such as environmental proximity to pollutants, genetic predisposition, and race/ethnicity have been shown to affect the onset of asthma. A recent review shows that the average prevalence of asthma among children in the United States is approximately 8%, while the prevalence within inner-city environments is 28% (Coleman et al., 2019). This is a health disparity, as most inner-city populations are primarily comprised of minorities with lower SES.

The United States Department of Health and Human Services reported that between the years of 2008 and 2010, the prevalence of asthma varied based on age, sex, and race (Akinbami et al., 2012). Recent findings reveal a sex bias in the prevalence and severity of asthma, which also varies by age group (Fu et al., 2014). In addition, racial differences have been noted, with a higher prevalence of asthma within African American populations when compared to Caucasians. Interestingly, there are ethnic differences in the prevalence of asthma within Hispanic population subgroups, with Puerto Ricans showing a 16.1% compared to 5.4% among Mexicans (Akinbami et al., 2012). Understanding racial and ethnic disparities within this population, for example, is further complicated by the “Hispanic paradox,” referring to the finding that Hispanic Americans tend to have better health outcomes compared to non-Hispanic White Americans despite being of lower SES (Franzini et al., 2001). It is important to note that the term “Hispanic” describes an admixed population with Spanish influence, typically including Native and African descendance; the degree and origins of admixture contribute to diversity within this population.

Several research groups have explored the role of ancestry on lung function in humans and using animal models (Brehm et al., 2012). Genome-wide association studies have identified genes associated with the development of asthma, with several genes reported to be involved in inflammation and immune function along respiratory airways. For example, *ORMDL3* overexpression in children was associated with asthma onset (Moffatt et al., 2010) and was later shown to increase airway remodeling in mice (Miller et al., 2014). In a Puerto Rican cohort, differential methylation was observed at sites located within genes involved in regulating respiratory airway integrity and function (*CDHR3* and *CDH26*) and immune response (*FBXL7, NTRK1*, and *SLC9A3*) (Forno et al., 2019).

A study by Miller and Chen (2006) showed a lowered bronchodilator response (BDR) and increased exposure to stress in Puerto Rican children. The study found that children with asthma who were exposed to chronic stress that was comparable to children without asthma displayed increased glucocorticoid receptor and β_2_-adrenergic receptor gene expression (Miller and Chen, 2006). CpG methylation patterns within *ADCYAP1R1* (receptor for adenylate cyclase), leading to polypeptide overexpression, are implicated in people suffering from posttraumatic stress disorder (PTSD) and anxiety (Chen et al., 2013). These combined findings were supported by a recent study in which a variant of *ADCYAP1R1* in combination with extrinsic stress led to reduced BDR in children with asthma and reduced levels of ADRB2 in CD4 + T cells (Brehm et al., 2015); the CD4+ cell type has been shown to have a significant role in an asthmatic response (Ling and Luster, 2016). Longitudinal epigenomic studies in different racial and ethnic groups investigating the development of asthma are needed. Studies of this nature may allow researchers to tease out associations between asthma prevalence and African ancestry, particularly in admixed populations such as Puerto Ricans.

### Midlife

#### Chronic Stress

Stress can exist in many forms ranging from biological stressors such as injury or illness to social stressors including low SES, fewer years of education, challenging relationship among family members, and neighborhood environments. Though acute stressors often do not pose a burden on health, situations in which an individual is chronically subjected to stress may have adverse effects on health and longevity (Schneiderman et al., 2005). Risk for several diseases has been associated with exposure to and duration of stress. Based on the 2006 Health and Retirement study, minority populations, such as African Americans and Hispanics, were reported to experience a greater stress burden when compared to Caucasians; these stressors are generally related to financial or housing situations (Brown et al., 2018). However, differences also exist in how certain subpopulations perceive and react to stress, with African American and Hispanics reported to be less likely to emotionally react to stressful situations in comparison to Caucasians (Brown et al., 2018).

Some of the disparities in stress response among different racial/ethnic subgroups may be due, in part, to biological vulnerability like genetic background and ancestry (Schneiderman et al., 2005). A comprehensive review by Argentieri et al. (2017) discusses how social and environmental stressors impact the risk for diseases such as hypertension, cardiovascular disease, cancer, and Alzheimer’s disease (AD) through epigenomic mechanisms. These studies demonstrated the role of differential DNA methylation in hypothalamic–pituitary–adrenal (HPA) axis genes, *CRH*, *CRH-R1/2*, *CRH-BP*, *AVP*, *POMC*, *ACTH*, *ACTH-R*, *NR3C1*, *FKBP5*, and *HSD11*β*1/2*, in different human diseases that exhibit health disparity. For example, differential methylation of HPA axis genes such as dehydrogenase *HSD11B1*, glucocorticoid receptor *NR3C1*, and chaperone *FKBP5* has been shown to be associated with environmental and social stressors such as childhood trauma, SES, and discrimination, as well as diseases such as hypertension, cancer, AD, and depression (Argentieri et al., 2017).

#### Addiction and Substance Abuse

Racial and ethnic disparities related to addiction and substance abuse are known to exist, though these disparities often show an abnormal trend due to combinatorial effects host genetics, health care, and SES. Based on data collected from the 12-year Northwestern Juvenile Project, non-Hispanic Whites, specifically males, were found to be at higher risk for drug abuse compared to African Americans and Hispanics (Welty et al., 2016). However, availability and/or affordability of addiction treatment may also contribute to this disparity. Failure to complete or enroll in addiction treatment for either drug or alcohol is frequently attributed to lack of health care, low SES, and unemployment. These conditions are commonly linked to African American, Hispanic, and Native American racial/ethnic groups (Saloner and Le Cook, 2014). In addition, emotional stressors and social adversities may also contribute to changes in epigenetic patterning, which in turn play a role in determining the type of response (positive or negative) an individual may have to a substance (Irner et al., 2012).

Epigenetic alterations have been linked to addiction and substance abuse. Studies by Kogan et al. (2018) have shown increased DNA methylation of oxytocin receptor *OXTR* in relation to stress, substance abuse, and high-risk sexual behavior in African American juveniles (∼19–23 years of age at baseline). *OXTR* is known to play a key role in buffering of stress responses and has been shown to be associated with increased stress and substance abuse symptomatology in young African American males (as demonstrated in the African American Men’s Project) (Kogan et al., 2018). Change in methylation status at *OXTR* was shown to be attributed in part to early-life adversity such as lack of prosocial ties. This gives support to the idea that social environments earlier in life are capable of shaping DNA methylation profiles leading to susceptibility to substance abuse in adolescence and mid-life stages.

Another layer of disparity in the context of addiction and substance abuse has been shown at the level of sex. An isolated study showed significant differences in gray matter volume within the brain between sexes in response to cigarette smoking (Wetherill et al., 2015). Sex differences in response to nicotine have also been observed, with females being less sensitive to nicotine, while males show greater reward responses to nicotine (Perkins et al., 2018). Females also appeared to be more responsive to varenicline, a pharmaceutical drug often used to treat smoking addictions (McKee et al., 2016). This emphasizes the importance of studying sex differences within ethnic subgroups in order to fully understand how individuals respond to a substance and to identify effective therapeutic strategies for treating substance abuse/addictions.

#### Metabolic Syndrome

Another common age-related condition that impairs health particularly in developed countries such as the United States is metabolic syndrome (MetS). This condition is characterized by insulin resistance, high fasting triglycerides, low high-density lipoprotein (HDL) cholesterol, incidence of hypertension, and obesity. Major disparities exist in MetS, with the highest prevalence observed among African Americans and Hispanic Americans (Ervin, 2009; Karlamangla et al., 2010; Heiss et al., 2014). In addition to disparity at the level of race/ethnicity, disparities also exist between sexes; for example, Mexican American females have a higher prevalence of MetS compared to males (Heiss et al., 2014). This sex-based disparity may be attributed in part to the significant differences in biological measures commonly used to diagnose the condition (Pradhan, 2014). The heritable risk of MetS is approximately 30%; however, it is considered to be largely a disease of lifestyle, with diet and exercise serving as important risk factors (Povel et al., 2011). Due to the significant involvement of environmental factors in determining MetS risk, many studies have explored the epigenetic mechanisms involved in MetS etiology/pathology.

Epigenetic mechanisms such as DNA methylation have been implicated in risk for MetS and related conditions. Racial/ethnic disparities in metabolic phenotypes do exist, as shown by the disproportionate prevalence of MetS in Mexican Americans. Groups have identified differentially methylated CpG sites in peripheral blood mononuclear cells (PBMCs) significantly associated with MetS, obesity, and hypertriglyceridemic waist (HTGW) (i.e., high waist circumference and elevated serum triglyceride concentration) in individuals of European and Hispanic ancestry (Ali et al., 2014; Mamtani et al., 2016). In Caucasian populations, methylation of *SOCS3*, which plays key roles in leptin and insulin signaling, has been shown to be significantly associated with MetS and other metabolic-related measures (as shown in participants enrolled in the Take Off Pounds Sensibly Family Study of Epigenetics) (Ali et al., 2014). Interestingly, in the same study, the degree of *SOCS3* methylation was inversely correlated with *SOCS3* expression, whereby hypermethylation led to declines in gene expression (Ali et al., 2014), suggesting a molecular explanation for the physiologic dysregulation observed in individuals with MetS. In Mexican Americans, differential methylation of *CPT1A* and *ABCG1*, involved in long-chain fatty acid and triglyceride metabolism, respectively, was found to be significantly associated with HTGW (as shown in participants of the San Antonio Family Heart Study) (Mamtani et al., 2016).

This emphasizes the importance of future studies to explore the epigenetic mechanisms underlying pre-MetS conditions such as obesity and bioenergetics dysregulation. Carless and colleagues have proposed to study to identify DNA methylation associated with several physiological measures of energy homeostasis and obesity utilizing the Viva la Familia (VIVA). This study was addressed to underpin the biological relevance of the onset and progression of metabolic disorders to tissues involved in the bioenergetics processes.

This avenue of research is not just important for developing future treatment and prevention strategies for combating MetS in individuals of different ancestral backgrounds. Metabolic dysregulation is also a well-known comorbidity factor for several age-related conditions that also display racial/ethnic health disparity (Brown et al., 2018). These conditions include cardiovascular disease, cancer, and neurodegenerative disease such as AD (Alzheimer’s Association, 2019).

### Late Life

Aging is a complex process whereby overall physiological functions decline over time, progressively influencing an individual’s susceptibility to disease. How an individual ages can provide information on driving factors that maintain a healthy state. Though genetic factors may partially determine human longevity (∼20–30%) and health, epigenetics serves as a meaningful bridge between genotype and phenotype, allowing us to identify how experiences and lifestyle affect aging and risk for disease (Pal and Tyler, 2016). A number of epigenetic modifications have been studied in humans and experimental animal models ranging in biological complexity (Pal and Tyler, 2016). These modifications are known to be dynamic throughout the life span (both in dividing and non-dividing cells), and changes to different epigenetic marks occur throughout the aging process, though the directionality of these modifications is dependent upon the genomic context. Further, advanced age is an important risk factor for a number of complex diseases; however, chronological age is not always informative of the true biological condition or disease risk of an individual. In recent years, epigenetic signatures such as DNA methylation have been investigated as biomarkers for predicting morbidity and mortality risk (Hannum et al., 2013; Horvath, 2013; Levine et al., 2018; Lu et al., 2019).

#### Cancer

Several types of cancers exhibit health disparities in the context of sex, race/ethnicity, SES, and geographic location. Throughout the literature, it is apparent that both histone and DNA modifications are associated with tumorigenic processes and may serve a therapeutic potential (Wee et al., 2014). Several clinical trials have revealed that therapies targeting histone and DNA modification can be effective in treating cancers. However, how these epigenetic modifications control metastasis and recurrence of cancers still remains an open area of study. Here, we broadly discuss the role that epigenetics may play in driving cancer disparities and the potential utility of epigenetic-based therapies targeting “readers,” “writers,” and “erasers.” For the purposes of brevity, we focus on colorectal cancer (CRC), colon cancer, and cervical cancer.

Though several types of cancer exist, cervical and CRC models have been used to demonstrate how social factors affect epigenetic patterning and impact racial cancer disparities. Racial/ethnic disparities in cervical cancer incidence and mortality rates are well documented. Cervical cancer mortality rates are twice as high in African American populations compared to non-Hispanic whites in the United States (Rates, 2007; Downs et al., 2008). Furthermore, non-Hispanic white women are more likely to be diagnosed at an earlier stage of cervical cancer than African Americans, Native Americans, or Hispanics (Gilliland et al., 1998; Del Carmen et al., 1999; Rates, 2007; Downs et al., 2008). Both incidence and mortality of CRC are higher among African Americans when compared to all other racial and ethnic groups (Gillette and Hill, 2015). Rates of CRC were reported to decline following the introduction of new prevention and screening methods such as at-home fecal occult blood test (FOBT) and increases in the recommended frequency of colonoscopies. Despite these efforts, the disparity failed to disappear, highlighting the multifactorial nature of the disease (Siegel et al., 2013). Differential epigenetic modifications may underlie these cancer disparities; research within the field has been advancing in recent years (Nebbioso et al., 2018).

Age-related cancers are often highly heterogeneous and arise due to combined interaction of genetic, environmental, and lifestyle factors. Several research groups have sought to understand how epigenetic changes are implicated in cancer etiology (Jones and Laird, 1999; Feinberg, 2004). One such example is cervical cancer, which has been linked extensively with human papillomavirus (HPV) 16 infection. Following HPV infection, two oncogenic proteins, early proteins 6 and 7 (E6, E7), activate the cell cycle growth and prevent cellular apoptosis, thereby allowing for accumulation of DNA damage (Graham, 2017). The mechanism involved in the etiology of cervical cancer involves the binding of E6-associated protein with ubiquitin ligase and deregulation of apoptosis *via* p53 leading to cellular proliferation and cancer (Graham, 2017).

The combined effects of HPV infection, smoking, and alcohol consumption have been shown to play a role in cervical cancer risk and disparity. American Indian (AI) women are known to smoke at rates four times greater than Caucasian women and most HPV-positive AI women are smokers (Karuri et al., 2017). Schmidt-Grimminger et al. (2011) have found that the carcinogen benzo[a]pyrene (BaP), commonly found in cigarette smoke, increases the expression of HPV oncogenes (E6/E7), suggesting that HPV infection and smoking may increase the incidence and severity of cervical cancers in AI women; this may account for some of the disparities in cervical cancer incidence and diagnosis between AI women and Caucasian women (Bell et al., 2011).

Targeting of epigenetic “readers,” “writers,” and “erasers” has been proposed as a therapeutic strategy for cancer treatment. However, target specificity toward tumorigenic vs. normal tissue and pharmacokinetic efficacy are important considerations due to the potential pleiotropic effects on cellular functions (Rajendran et al., 2019). Many therapeutic strategies for cancer treatment have largely been focused on targeting “writers” and “erasers.” Thus far, two classes of epigenetic drugs have been approved by the United States Food and Drug Administration, DNMT and HDAC inhibitors, with other targets in late-stage clinical trials [e.g., bromodomain and extra-terminal (BET) inhibitors (BETis)] (Roberti et al., 2019). Interest in the use of natural substances as epigenetic therapies for certain cancer subtypes has increased in recent years as a result of their potential to be more effective chemopreventive and chemotherapeutic strategies (Meeran et al., 2010). One example is the compound sulforaphane (SFN), an isothiocyanate found in cruciferous vegetables. SFN may serve as an efficient, more accessible, and affordable anticancer agent. SFN and its associated metabolites have been found to act as natural HDAC inhibitors, through their propensity for acetylation activity on *CCAR2-encoded protein* (cell cycle and apoptosis regulator 2) (Parnaud et al., 2004). Overexpression of *CCAR2* has previously been correlated with poor survival outcomes in colon cancer (Clarke et al., 2008; Best et al., 2017). Human clinical trials provide strong evidence for the chemopreventive effects of SFN on carcinogenesis by preventing tumor growth and increasing sensitivity of cancer cells to chemotherapy (Jiang et al., 2018). SFN has been found to induce apoptosis of tumors in a mouse model through inhibition of HDACs (Jiang et al., 2018). SFN has also been shown to significantly decrease the expression of DNMTs through allowing for modulation of cyclin D2 expression ultimately promoting pancreatic cancer cell death (Jiang et al., 2018). New and effective treatments, nutritional adaptations such as Nano-Curcumin (Nano-CUR) and food items enriched with SFN that target epigenetic regulatory mechanisms, may help diminish gaps in cancer-related health disparities.

Another natural substance proposed as an epigenetic therapy for cancer is curcumin. Curcumin has been shown to downregulate HPV18 transcription and exhibit enhanced cytotoxic activity in HPV-infected cells (Zaman et al., 2016). Furthermore, Nano-CUR, a nanoparticle formulation designed for increased absorption, was shown to abrogate the expression of BaP-induced E6/E7 (Zaman et al., 2016). In the context of CRC, oncogenic LncRNA *MALAT1* can be used as an indicator of poor prognosis (Xu et al., 2018). Analysis using The Cancer Genome Atlas (TCGA) demonstrated a higher expression of *MALAT1* in African American CRC tissue compared to Caucasians. Thus, *MALAT1* represents a marker for the disparate CRC incidence and severity in African Americans and Caucasians. Nano-CUR has been proposed as an effective epigenetic treatment modality for HPV-based CRC and, more selectively, cervical cancers in populations with greater rates of smoking without the same toxicity as current anticancer therapies including chemo and radiation therapy regimens.

Most research to date has focused on targeting “writers” and “erasers” as therapeutic strategies for epigenetic cancer treatment. The discovery of BETis has resulted in an increased focus on targeting chromatin modification “readers.” Recent studies have shown that two BETis are effective in the downregulation of the *MYC* oncogene in several cancer subtypes, suggesting the importance of these inhibitors in oncogenic regulation and for cancer therapies (Filippakopoulos et al., 2010; Dawson Mark and Kouzarides, 2012). The role of BETis in cancer stems from their interference with the cancer cell cycle progression and DNA repair (Mio et al., 2019). Multiple BETis have shown promise for their therapeutic effects across several subtypes of cancers (Sahai et al., 2016). As such, BETis use has begun in clinical trials, providing another target for epigenetic cancer treatment. Preliminary clinical trials have revealed the effectiveness of BETis in cancer therapy. One benefit of their use is that they are unable to singularly bind a bromodomain-containing family member, which could reduce their therapeutic side effects (Simó-Riudalbas and Esteller, 2015; Ronnekleiv-Kelly et al., 2017). However, further research is needed to investigate “readers” dysfunction in cancer and identify the chemical compounds and probes capable of inhibiting “readers” (Mio et al., 2019).

#### Alzheimer’s Disease

Alzheimer’s disease is a multifactorial disease with both genetic and lifestyle factors impacting risk. Though early-onset forms of the disease display an inheritance pattern that is more Mendelian in nature, understanding the etiology of late-onset AD (LOAD) proves rather difficult due, in part, to the issue of “missing heritability.” This concept refers to the limited contribution that genetic variants have in explaining the heritable risk of LOAD. Because of this, several groups have begun to explore the role of epigenetics and the different environmental and lifestyle factors that may contribute to AD risk.

It is known that major disparities exist in AD prevalence and pathology across race/ethnicity, with Hispanics and African Americans having 1.5 and 2 times increased risk, respectively (Gaugler et al., 2019). Within the United States, the incidence of AD is known to be especially elevated in Hispanic and African American females (Matthews et al., 2019). Though genetic factors may explain some of this disparity, a great deal is thought to be due to external factors such as diet, lifestyle, and physical environment. Risk for AD is already known to be higher in individuals of advanced age, lower SES, and/or who suffer from a comorbid condition such as obesity, MetS, and hypertension (Gaugler et al., 2019).

Some research groups have taken an innovative approach toward understanding AD risk by determining how early life exposures (as early as neonatal stages) to extrinsic (e.g., metal toxicity), intrinsic (e.g., cytokines, hormones), and dietary factors (e.g., nutrient imbalance) impact gene expression and ultimately physiological development and function. Lahiri et al. (2007) have proposed the Latent Early-life Associated Regulation (LEARn) model of AD that early life exposures, which are determined in part by social disparities, can disrupt gene regulation, though this perturbation does not become pathogenic until later stages in life. This model is similar to the “two-hit hypothesis” for cancer, whereby an initial insult alters gene expression, followed by a latent period; if a second insult arises later in life, the alteration then becomes aberrant. It further proposes that overproduction of amyloid precursor protein (APP) and amyloid beta in late life may be triggered by early-life changes to the methylation status of promoters for *APP* and other related genes (Lahiri et al., 2007). Their group has also proposed epigenetic therapies such as mithramycin and tolfenamic acid to target amyloid pathways; these have been shown to exhibit downstream impact on neuronal structures including cell body, neurite length, and branch points (Bayon et al., 2017). Though AD is an age-related disease, emphasis must be placed on further understanding how early-life and mid-life environments and exposures influence AD risk in later-life stages.

## Challenges and Current Limitations of Social Epigenomic Studies

It is important to acknowledge that studies within the field of social epigenomics are often met with several challenges and limitations; this includes adequate study design, sample availability, experimental techniques, statistical analysis, and biologic interpretation of results (Bakulski and Fallin, 2014). Though social epigenomics may be useful in addressing racial/ethnic disparities in the context of health and disease, there is a lack of epigenetics literature investigating minority populations, with a great majority of studies focusing on homogeneous populations (i.e., Caucasians). This absence is primarily due to limited availability of samples from minority groups, which can be attributed to biased sampling and difficulty in recruiting/retaining research subjects. Much of the past literature has been focused on clinically recruited populations, which introduce bias and are problematic, specifically for studies investigating the effects of different social/environmental factors. One such example is Berkson’s bias, whereby clinic attendance is impacted by exposure and/or accessibility to clinical settings and presence of pre-existing diseases and/or conditions, resulting in distortion of experimental findings (Westreich, 2012). For example, evidence suggests that individuals belonging to minority populations hold mistrust toward researchers and health care personnel in fear of being exploited or mistreated (Yancey et al., 2006). Efforts to mitigate this mistrust have been made by some groups, primarily through improving communication and becoming more involved in the community (Yancey et al., 2006). Additionally, in most population-based studies, race is often self-reported, which does not capture the biological ancestry of an individual. Ancestry-informative markers (AIM) in the form of single-nucleotide polymorphisms (SNPs) provide utility in further defining population structure to allow for a more comprehensive understanding of the molecular factors associated with certain health disparities.

With epigenetic changes occurring throughout the course of an individual’s lifetime, it is difficult to capture the full effect of epigenetic changes on risk of disease. It is important to consider that any measurement is only a snapshot in time of a reversible modification, which further complicates biological interpretation. Samples collected at the time of disease diagnosis do not allow researchers the luxury of assuming a role for epigenetic changes in disease onset. Access to longitudinal data or sampling can be useful in teasing out some of the biological changes occurring downstream of epigenetic modifications; however, generation of this type of data is time-consuming and quite costly. Furthermore, depending on the epigenetic modification in question, sample storage can also greatly impact experimental results. Stability of different epigenetic modifications varies across sample conditions. For example, DNA methylation remains relatively stable in frozen tissue, while chromatin analysis necessitates fresh tissue (Bakulski and Fallin, 2014). Additionally, tissue samples are highly heterogeneous in nature and contain several cell types; adjustment for the proportions of each cell type whether by experimental methods (i.e., flow cytometry; limited to fresh tissue) or statistical methods (i.e., cell type correction) is essential for deconvoluting the effects of cell type on epigenetic modifications (Bakulski and Fallin, 2014). High-throughput methods also demand multiple batches to be analyzed, which need to be corrected for in the analysis stage of these studies (Bakulski and Fallin, 2014). The biologic interpretation of results from epigenomic studies remains a challenge, specifically, understanding the direct mechanisms for how various exposures cause epigenetic changes and lead to disease.

Difficulties often arise in teasing out gene–environment interactions. Genetic variants (i.e., SNPs) have been associated with racial/ethnic health disparities. For a recent review on the utility of investigating genetic data in the context of health disparities, refer to Mersha and Abebe (2015). Importantly, SNPs have also been associated with altered epigenetic modifications. For example, genotypes at certain loci have been shown to result in differential patterns of methylation (Smith et al., 2014); these positions are otherwise referred to as methylation quantitative trait loci. Since SNPs can affect both intermediate (i.e., epigenetic modifications) and downstream (i.e., conditions/diseases) phenotypes, complex analysis strategies must be used to tease apart these relationships. The use of statistical and epidemiological principles in testing causal associations presents some promise for epigenomic research. Directed acyclic graphs (DAGs) can be used to visually explain the potential direct and indirect causal mechanisms between exposure and outcome, as well as identify potential factors for mediation analysis (Mueller et al., 2020). For example, environmental exposures can lead to epigenetic changes, affecting disease risk downstream. Testing mediation effects between exposures and corresponding epigenetic changes can help identify mechanisms that mediate the exposure–outcome relationship (VanderWeele, 2016; Gao et al., 2019). Unfortunately, mediation analysis does have its limitations, the use of mediation requires a large sample size, oftentimes unavailable in epigenetic studies. An alternative strategy is Mendelian randomization (MR) which utilizes genetic variants as proxies for a valid instrumental variable for estimation of the causal effect between an exposure and outcome, overcoming confounding and reverse causation (an issue common to epigenetic research) (Smith and Ebrahim, 2003; Relton and Davey Smith, 2015). Another statistical method for elucidating causal inference is inverse probability of treatment weighting (IPTW), a form of propensity weighting for inclusion in statistical analysis. IPTW is useful in controlling for selection bias in epigenetic studies. Within the context of epigenomic research, this statistical method relies on creating a propensity score and inverts the score to weigh individual covariates in order to estimate the level to which epigenetic changes would exist if different racial or ethnic groups experienced similar built and social environments (Beck et al., 2016). IPTW has proven useful in teasing out causation of racial disparities for some conditions. For example, in a study adjacent to the Greater Cincinnati Asthma Risks Study, 695 African American and White children from an urban pediatric hospital were studied to identify potential asthma onset predictors (Beck et al., 2016). Preliminary results showed racial disparities in exposure to risk factors including allergen sensitization and socioeconomic hardships. However, upon further examination using IPTW-adjusted survival analysis controlling for a number of exposures, the risk for readmission for asthma was comparable between groups. This suggests that methods such as those mentioned above may be useful for socio-epigenomic researchers in determining disease causality.

## Conclusion

This review article has focused on the social epigenetic mechanisms that may lead to chronic diseases and resulting health disparities in specific populations. The review draws from transdisciplinary sciences encompassing basic research, public health, and medicine, as well as community organizations, to highlight the current state of knowledge, future directions, and the challenges and limitations of socio-epigenetics research. Understanding the impacts that environment and lifestyle factors have on biological processes and how these factors can be modified to improve the state of health on a global scale is the primary goal of social-epigenetic research. We have discussed the ways in which social and environmental factors impact biological processes through epigenetic changes leading to susceptibility to certain conditions and/or diseases throughout different life stages (Figure 1) and how these changes contribute to health disparities. Approaches which may help in mitigating the complex health disparities impacted by epigenetic-related mechanisms were highlighted including pharmaceutical targeting of epigenetic imprinting, adaptations to specific nutrition/diet-based therapy like consumption of cruciferous vegetables, improvements to our local environments like creation of increased green space and related community infrastructure, and psychosocial practices. We also addressed current challenges and limitations in social epigenomics research and highlighted the need for more minority population-based cohorts in social epigenomic studies.

## Author Contributions

VM, NP, and TS: review and manuscript writing. RB, MF, HJ, NP, M-HT, and ST: supervision, administration, and manuscript review. JV: conceptualization, funding, administration, and manuscript review. All authors contributed to the article and approved the submitted version.

## Conflict of Interest

The authors declare that the research was conducted in the absence of any commercial or financial relationships that could be construed as a potential conflict of interest.
